# Use of CRISPR Technology in Gene Editing for Tolerance to Biotic Factors in Plants: A Systematic Review

**DOI:** 10.3390/cimb46100659

**Published:** 2024-10-02

**Authors:** Marcelly Santana Mascarenhas, Fernanda dos Santos Nascimento, Anelita de Jesus Rocha, Mileide dos Santos Ferreira, Wanderley Diaciso dos Santos Oliveira, Lucymeire Souza Morais Lino, Tiago Antônio de Oliveira Mendes, Claudia Fortes Ferreira, Janay Almeida dos Santos-Serejo, Edson Perito Amorim

**Affiliations:** 1 Department of Biological Sciences, Feira de Santana State University, Feira de Santana 44036-900, BA, Brazil; marcelly.bio@hotmail.com (M.S.M.); diacisowanderley@hotmail.com (W.D.d.S.O.); 2 Embrapa Mandioca e Fruticultura, Cruz das Almas 44380-000, BA, Brazil; feel.20@hotmail.com (F.d.S.N.); anelitarocha@gmail.com (A.d.J.R.); mileideferreira12@gmail.com (M.d.S.F.); lucymeire.lino@gmail.com (L.S.M.L.); claudia.ferreira@embrapa.br (C.F.F.); janay.serejo@embrapa.br (J.A.d.S.-S.); 3 Department of Biochemistry and Molecular Biology, Federal University of Viçosa, Viçosa 36507-900, MG, Brazil; tiagoaomendes@ufv.br

**Keywords:** biotic stress, CRISPR/Cas, plant diseases, phytopathogens, pests, plant genetic improvement

## Abstract

The objective of this systematic review (SR) was to select studies on the use of gene editing by CRISPR technology related to plant resistance to biotic stresses. We sought to evaluate articles deposited in six electronic databases, using pre-defined inclusion and exclusion criteria. This SR demonstrates that countries such as China and the United States of America stand out in studies with CRISPR/Cas. Among the most studied crops are rice, tomatoes and the model plant *Arabidopsis thaliana*. The most cited biotic agents include the genera, *Xanthomonas, Manaporthe, Pseudomonas* and *Phytophthora*. This SR also identifies several CRISPR/Cas-edited genes and demonstrates that plant responses to stressors are mediated by many complex signaling pathways. The Cas9 enzyme is used in most articles and Cas12 and 13 are used as additional editing tools. Furthermore, the quality of the articles included in this SR was validated by a risk of bias analysis. The information collected in this SR helps to understand the state of the art of CRISPR/Cas aimed at improving resistance to diseases and pests to understand the mechanisms involved in most host–pathogen relationships. This SR shows that the CRISPR/Cas system provides a straightforward method for rapid gene targeting, providing useful information for plant breeding programs.

## 1. Introduction

Biotic stresses caused by pests and pathogens such as viruses, bacteria, fungi, oomycetes, nematodes, and insects are largely responsible for low productivity in various crops [1]. In addition, the continuous increase in several new pest species makes the control of these pathogens challenging [2]. Microorganisms have specific characteristics and are classified into groups. Biotrophic microorganisms depend on the living plant to feed and complete their life cycle; necrotrophs, during their feeding habit, kill the host plant, and hemibiotrophs initially depend on the living plant (behaving like biotrophs) in order to survive and complete their cycle with a necrotrophic phase; where the host is degraded [3,4].

The plant and the pathogen are intertwined in a battle of recognition and evasion where a multilayered defense system, including pathogen-associated molecular pattern (PAMP)-triggered immunity (PTI) and effector-triggered immunity (ETI), has evolved in plants to fight invading pathogens for survival [5]. In general, PTI uses pattern recognition receptors to monitor PAMPs on the cell surface. Meanwhile, ETI relies on leucine-rich repeat receptors with a nucleotide-binding domain to recognize pathogen effectors inside the cell [6,7,8].

Thus, understanding the molecular mechanisms of pathogen–host interactions, especially the identification of key targets related to defense responses in plants, would offer a great opportunity to design broad-spectrum and durable resistance in various crops [5,9,10]. Hence, plant breeding programs are looking for effective and long-lasting techniques to improve crops. However, some challenges, such as the complex inheritance of the vast majority of agronomic traits and the strong genotype–environment interaction, are still challenging [11].

Currently, three types of genome editing tools are widely used by researchers, including zinc finger nuclease (ZFN) [12], transcription activator-like effector nuclease (TALEN) [13], and CRISPR-clustered/associated regularly interspaced short palindromic repeats (CRISPR/Cas) [14]. ZFN and TALEN have not been widely used due to high costs and failures. The CRISPR/Cas system (which includes Cas9, Cas12, and Cas13) from a prokaryotic organism has transformed the field of gene editing with high efficiency and easy handling and application. Compared to the previous two generations of genome editing techniques, the CRISPR/Cas system is flexible, simple, stable, and easy to transform. These resources allowed for ZFN and TALEN to be replaced by CRISPR/Cas, which has become one of the main genome editing techniques.

CRISPR is composed of CRISPR RNA (crRNA) (transcribed from the spacer sequences) and transactivating crRNA, or single chimeric guide RNA (sgRNA) (formed by the fusion of crRNA and tracrRNA) for targeting and the specificity of targeting [14,15]. The Cas9 protein-RNA complex (from *Streptococcus pyogenes*) is formed by the combinations of the crRNA spacer to a target sequence close to an adjacent motif of the proto-spacer (PAM—3 base pair (bp) motifs essential for spacer acquisition and target cleavage) [15,16,17].

Due to its ease of execution, the CRISPR/Cas system has become the tool of choice for gene editing in any species of interest. By generating a double-strand break (DSB) at the desired site by the Cas-gRNA complex, the host–cell repairs the DNA lesion via the non-homologous end joining (NHEJ) pathway, resulting in short insertions or deletions, consequently leading to gene knockouts. Another form of repair is the homology-directed repair (HDR) pathway, which is more precise and has a lower probability of error [18,19]. In plants, the system has been used to knock out all members or a single member of a multigenic family [20] and even several unrelated genes [21], with the NHEJ pathway being the most reported [22].

Several studies have been published to demonstrate the different genes that positively or negatively regulate resistance to various pests and pathogens in model plants and diverse crops, such as *Arabidopsis thaliana*, where genes such as *ZAR1*, *UGT71C3*, and *miR398b* have been studied [23,24,25], in rice, *SWEET14*, *eIF4G*, and *PRAF2* [26,27,28], in maize, *ZmACD6*, *Zmksl2*, and *JAZ15* [29,30,31], in tomato, *SlWRKY16*, *SlWAT1*, and *SlDMR6* [32,33,34], and in soybean, *Rfg1*, *Rpp1*, and *GmLMM1* [35,36,37].

In addition, CRISPR technology has evolved rapidly and has shown great potential for plant biology, especially with regard to CRISPR/Cas9 variants, such as CRISPR/Cas12 and CRISPR/Cas13, which offer better specificity for DNA and RNA, respectively. For precise changes in a single base, without causing double-strand breaks, reducing off-target mutations, base editing methods have been implemented [38,39]. Other improved delivery tools, such as the use of nanoparticles and viral vectors, allow for the efficient introduction of the CRISPR system into plant cells and the delivery of RNP (ribonucleoprotein) complexes, rather than DNA plasmids, is being used to improve efficiency and reduce off-target effects [40]. Other advanced techniques allow for the simultaneous editing of multiple sites in the genome, making it easier to modify multiple traits at the same time, and systems such as Prime Editing and CRISPR 3.0 are emerging, allowing for precise insertions and deletions without the need for DNA breaks [38,39,41].

Thus, off-target effects have been significantly reduced due to improvements in the specificity of the CRISPR/Cas system. In addition, these data facilitate reliability and safety, allowing for regulatory approval, coupled with strategies such as temporary editing, where CRISPR machinery is rapidly degraded after editing, as well aa the ability to edit multiple genes simultaneously, which facilitates the engineering of complex traits in plants, such as disease resistance and nutritional trait improvements [39,40,41,42].

In order to systematically gather and review current research on the use of CRISPR/Cas technology in gene editing for biotic stress tolerance, this study presents a systematic review (SR) of articles published in the last twelve years. It also aims to contribute to the SR previously carried out on the use of CRISPR/Cas technology in gene editing for tolerance to abiotic stresses [22]. Here, we describe how the technique has been applied to pest and pathogen resistance studies and the locations and crops, among other data, for which it is possible to detect the current research trend on the subject and its impact on crops.

## 2. Materials and Methods

To carry out this SR, the State of the Art through Systematic Review (StArt) software (version 3.0.3 Beta) was used, developed, and made available by the Software Engineering Research Laboratory of the Federal University of São Carlos, at https://www.lapes.ufscar.br/resources/tools-1/start-1, accessed on 15 August 2023.

The review was prepared following the Preferred Reporting Items for Systematic Reviews and Meta-Analysis (PRISMA) guidelines [43], structured in a set of evidence-based items that help authors report a wide range of systematic reviews and meta-analyses and can be used in plant, animal, and health intervention areas. A PRISMA checklist was drawn up to minimize bias in this SR, available at https://doi.org/10.5281/zenodo.13869284 (accessed on 29 September 2024). The SR process using StArt occurred in three stages: planning, execution, and summarization.

### 2.1. Planning

In order to plan the SR, a protocol was developed, available at https://doi.org/10.5281/zenodo.13371943 (accessed on 26 August 2024), which includes a description of the SR, the research objectives, the main/guiding question, the research questions (Table 1), the search string, the source mechanism, the inclusion and exclusion criteria, and the definition of the types of study. The question guiding the SR was based on the Population Intervention Comparison Results strategy [44] (Table 1). Thus, this SR aims to answer the following research question: how has CRISPR/Cas technology been used in gene editing in plants for biotic stress tolerance over the last twelve years?

After drafting the main research questions, secondary questions were elaborated (Table 2).

### 2.2. Execution

Searches were performed in different electronic databases such as Pub Med Central, Springer, Scopus, Web of Science and sites such as Google Scholar and CAPES Periodicals Portal. For the Google Scholar, Springer, PubMed Central, CAPES Periodicals, Web of Science and Scopus databases, the following keywords were used: (“CRISPR/Cas9” OR “CRISPR-Cas9” OR “CRISPR-Cas in plants”) AND (“plant resistance” OR “plant disease resistance”) AND (“plant disease” OR “biotic factors” OR “disease resistance” OR “plant pathogens” OR “pests” OR “plant parasite”).

For the Web of Science and Scopus databases, another search string was also used, with the following keywords: (“CRISPR” OR “CRISPR/Cas9” OR “CRISPR-Cas9” OR “CRISPR-Cas in plants”) AND (“biotic factors” OR “pathogen resistance” OR “phytopathogen resistance” OR “plant disease resistance” OR “disease resistance” OR “plant resistance” OR “pest resistance” OR “parasite resistance”), seeking to include as many studies as possible.

The Boolean connectives “AND” and “OR” were used to differentiate search terms and group synonymous terms, respectively. The search results in each database were imported into the BIBTEX, MEDLINE, or RIS formats, compatible with the StArt software. The bibliographic survey was performed from January 2013 to July 2024.

To select the articles, the title, abstract, and keywords were analyzed. Articles that met the terms of the search sequence and did not deviate from the proposed theme were accepted and submitted to the extraction stage. At this stage, only articles that answered the research questions (Table 2) previously established in the SR protocol were accepted as an inclusion criterion. Exclusion criteria were also used to extract the following articles: theses, dissertations, manuals, book chapters, review articles, papers not written in English, papers without a clear contribution, papers published prior to 2013, or papers that were off-topic.

### 2.3. Data Summarization

The data obtained from the scientific articles was summarized in tables, graphs, word clouds, and bibliometric maps. The graphs were constructed using the R version 4.4.1 statistical environment [45], using the ggplot2, reshape2, and ggpubr packages. The bibliometric analyses were performed according to the metadata of the selected articles using the VOSviewer_1.6.17 program [46] to verify the networks of interactions between keywords and between authors and co-citations. Word clouds containing the journals used to publish the articles, genes edited, tools, and software used to support the CRISPR/Cas tool over the last twelve years were generated online and free of charge (https://www.wordclouds.com/, accessed on 18 November 2023), based on the frequency of the data.

### 2.4. Risk of Bias Analysis

To assess the risk of bias, the adapted Cochrane Collaboration Tool [47] was used. The methodological quality was analyzed by three authors (MSM, FdSN, and AdJR), and the articles selected in the extraction stage were subjected to four questions (Table 3) in order to further reduce data bias. These are essential questions that confirm whether editing using CRISPR/Cas was effective, reaching the target site or not.

Systematic errors in scientific studies that cause distortions in the results can happen; it is complex to state whether a study is biased or not, but systematic errors in scientific studies can be estimated and minimized through a careful evaluation of its methodological quality. Rigorous practices such as protocol development, the use of PICOS strategy, PRISMA checklist, and the others described above, significantly reduce the risks of bias.

The risk of bias can be classified as low, moderate, or high when the study presents negative responses (“no”) of up to 25%, between 25 and 75%, and greater than 75%, respectively.

## 3. Results

### 3.1. Bibliographical Survey

Initially, 9513 studies related to the proposed topic were identified from the search strings in the selected electronic databases, which are widely used in plants. Google Scholar showed 5880 studies, Pub Med Central showed 1421, CAPES Periodicals Portal showed 509, Scopus showed 819, Web of Science showed 574, and Springer, 310. Although the Web of Science and Scopus databases use two search strings, Google Scholar contributed 61.8% of the articles submitted, which is justified by its broad search spectrum. From this total, 376 were detected as duplicates by the StArt software.

**Figure 1:**
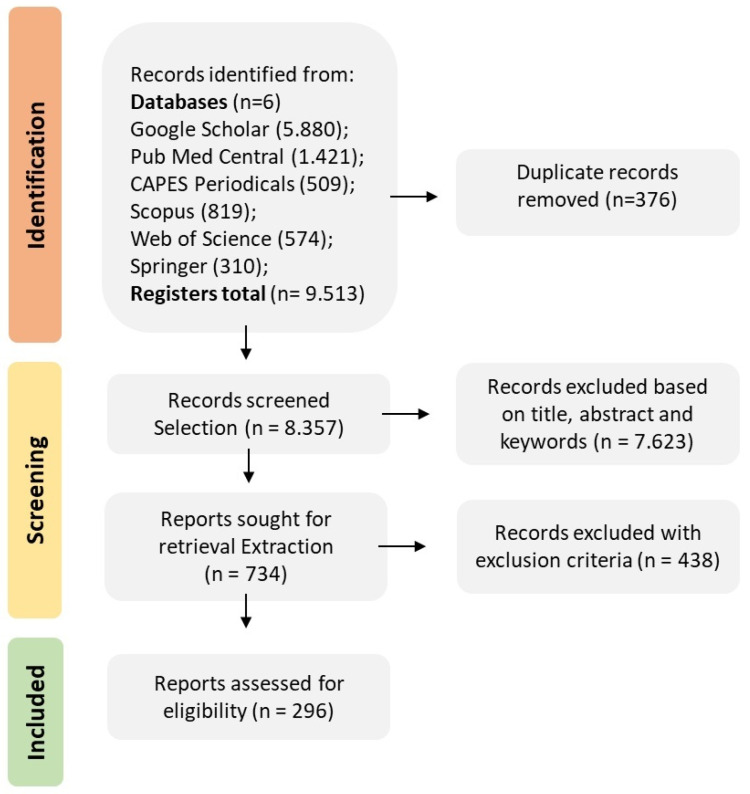
A PRISMA flow diagram with the respective stages of the process of selecting studies for inclusion/exclusion in the systematic review of the CRISPR/Cas technology used to edit genes for tolerance/resistance to biotic stress in plants according to the databases [43].

After analyzing the title, abstract, and keywords, 7623 studies were rejected and 734 were submitted to the extraction stage. The texts were read in full, resulting in 296 accepted articles (Figure 1). These selected articles met the inclusion/exclusion criteria because they are related to the theme of this SR, which aimed to include as many studies as possible on the use of CRISPR/Cas technology in the editing of tolerance/resistance genes to biotic stresses in the last 12 years; then, the information was deposited in the Supplementary Materials (Table S1) for consultation.

The studies evaluated covered the period from January 2013 to July 2024, with 2021 considered the year with the highest number of publications as to CRISPR/Cas technology in the editing of genes related to resistance to biotic stressors, contributing 21.6% of the articles. The other years had 16.6% (2022), 16.2% (2020), 11.5% (2023), 10.5 (2024), 9.8% (2019), 7.4% (2018), 3.7% (2017); for the years 2016, 2015, 2014 and 2013, less than 2% were obtained.

**Figure 2:**
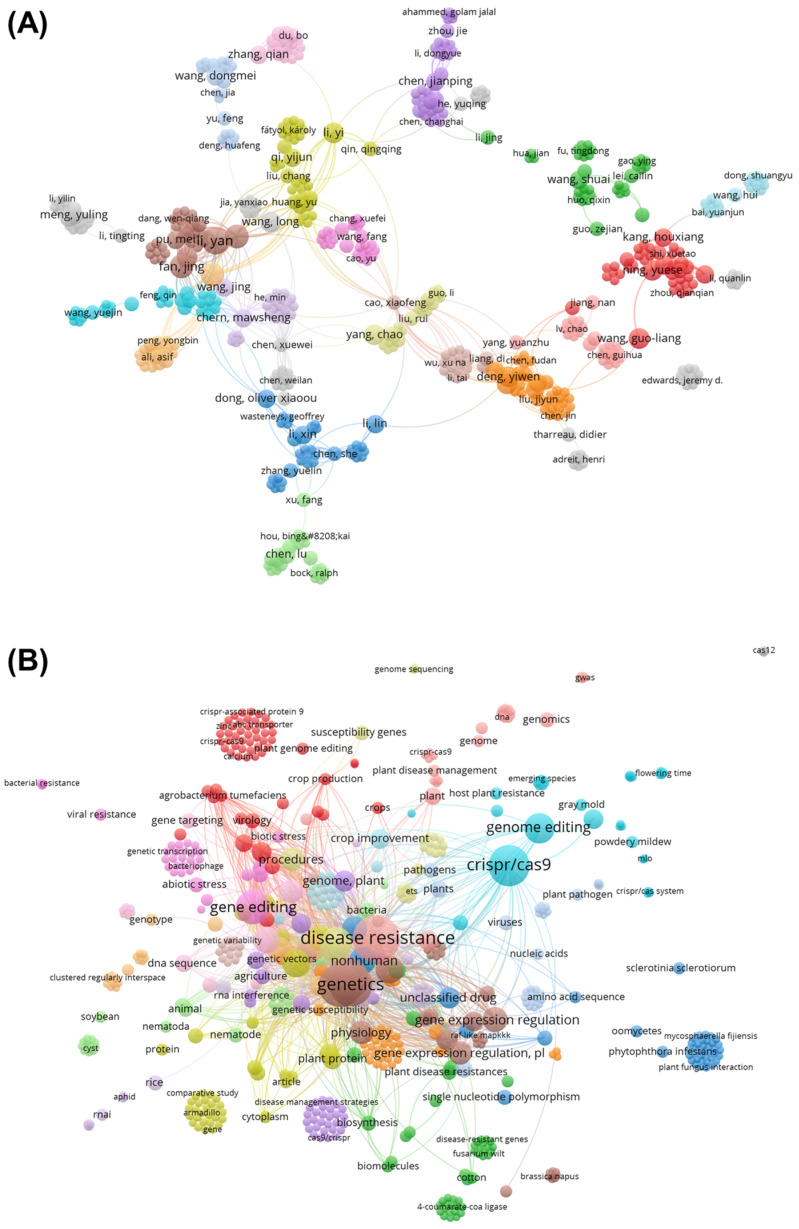
Bibliometric indicators of the collaboration network between authors and keywords of the selected articles on CRISPR/Cas technology and biotic factors. (**A**) Collaborators who have published the most on CRISPR/Cas and biotic stresses in the last 12 years. (**B**) Keywords of the selected articles on CRISPR/Cas technology used for gene editing of tolerance/resistance to biotic stresses in plants during the extraction phase of this systematic review. Different colors for each circle indicate collaboration between groups.

Considering the frequency of authors and all the keywords in the articles selected in the extraction phase, bibliometric maps were developed to represent the co-occurrence of these words (Figure 2). The size of the circles represents the number of times these words were repeated; the larger the circle, the more times the author and journal were cited. Colors indicate different groups of authors and keywords and the thickness of the lines the correlation between these words. The thicker the line, the higher the occurrence of the term.

Twenty clusters were formed and identified using different colors, according to the degree of similarity between the authors’ works (Figure 2A). Authors such as Yan Li, Jing Fan, Long Wang, Yuese Ning, Yi Li, Chao Yang, Liang Guo Wang, Qian Zhang, Jianping Chen, and Jing Wang are responsible for a large bibliographic contribution. These data demonstrate a trend in centralized research related to Chinese authors with not much exchange of information between Chinese researchers and those from the rest of the world. The links or distances infer the correlation between these authors and their collaboration on other works. Some small grouped but isolated nodes can be observed, but they show minimal contribution to the studies performed by the authors included in these groups.

For the keywords, approximately 92 nodes and 12 clusters were observed, which defined the main research themes in this area. The most relevant groups according to the size of each circle refer, in order of relevance, to the following words: disease resistance, genetics, CRISPR/Cas9, gene editing, and genome editing. These words form core groups associated with several other terms of collaboration with a theme that constitutes the smaller groups formed, for example, by the terms vectors, crop breeding and regulation of gene expression. Words such as Cas12, RNAi, bacterial resistance, and genomic sequencing appear in isolation, which indicates lower frequency and low correlation with other studies (Figure 2B).

### 3.2. Plant of Origin and Plant Cultures Edited Using CRISPR/Cas Technology

**Figure 3:**
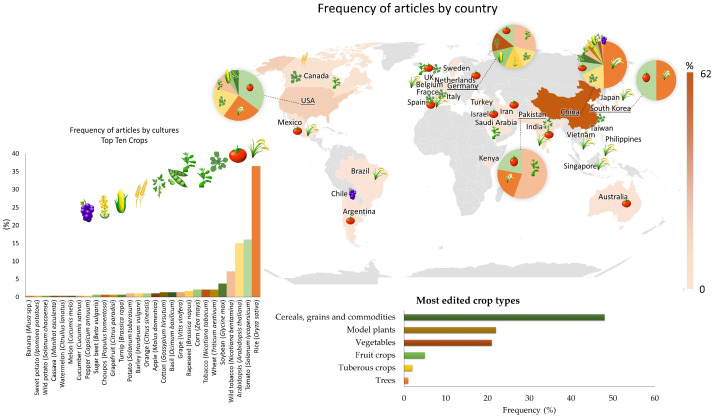
Frequency of articles according to country of publication and crop edited by CRISPR/Cas technology for plant disease tolerance/resistance. More than one plant species per article was considered in calculating the frequency.

Of the 296 research papers, 158 (59.8%) originated in China, 30 (11.4%) in the United States of America (USA), 12 (4.5%) in Germany, 7 (2.7%) in South Korea, 5 (1.9%) in Canada and Pakistan, 4 (1.5%) in Spain and Saudi Arabia, 3 (1.1%) in India, Israel, Japan, and the Netherlands, and 2 (0.8%) in Australia, the Philippines, Sweden and the United Kingdom. The other countries only had one article submitted, which represents just 0.4% of the publications on the subject (Figure 3).

China and the USA are the countries that produce and disseminate the most scientific knowledge on the subject. However, all continents, except for Antarctica, have contributed to this area of research. The articles identified 28 plant species used for gene editing related to resistance to biotic factors. Overall, the types of crops most edited by the CRISPR technique include cereals, grains and agricultural commodities, with 48% of the studies represented mainly by rice, followed by studies with model plants, represented mainly by *Arabidopsis*. Other studies have included vegetables (21%), fruits (5%), tubers (2%) and trees (1%) (Figure 3).

Rice (*Oryza sativa*) was the most studied crop, present in 36.5% (109) of the studies, followed by tomato (*Solanum lycopersicum*) with 16% (48), *Arabidopsis thaliana* with 15% (45), wild tobacco (*Nicotiana benthamiana*) with 7.2% (22), soybean (*Glycine max*) with 3.8% (12), wheat (*Triticum aestivum*), tobacco (*Nicotiana tabacum*), and corn (*Zea mays*), with 2% (6), rapeseed (*Brassica napus*) with 1.7% (5), and grape (*Vitis vinifera* L.), basil (*Ocimum basilicum*), and cotton (*Gossypium hirsutum*) with 1.4% (4). The other species had a frequency of less than 1% of the studies (Figure 3).

### 3.3. Biotic Stresses in Plants

**Figure 4:**
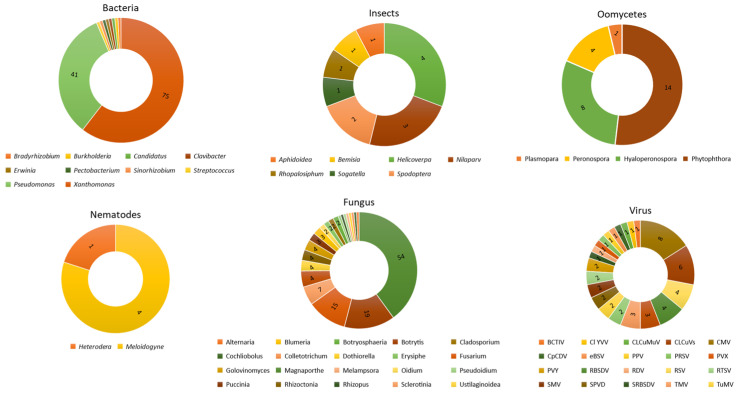
The most-studied biotic agents (bacteria, insects, oomycetes, nematodes, fungi, and viruses) in the last twelve years for resistance/tolerance to plant diseases using CRISPR/Cas technology. More than one biotic agent per article was considered in calculating frequency.

The biotic agents cited in the literature were bacteria, fungi, viruses, oomycetes, insects, and nematodes, accounting for 51.8, 28.3, 10.5, 5.7, 2.7, and 1%, respectively. The genera *Xanthomonas* (75) and *Pseudomonas* (41) account for 92% of the studies on bacteria. For fungi, the most studied genera were *Magnaporthe* (54), *Botrytes* (19), *Fusarium* (15), *Sclerotinia* (7), and *Verticillium* (6) (Figure 4).

The most-cited viruses were cucumber mosaic virus (CMV) (8), cotton leaf curl virus (CLCuVs) (6), rice streak virus (RSV), rice black-streaked dwarf virus (RBSDV) (4), tomato yellow leaf curl virus (TYLCV) (3), and tobacco mosaic virus (TMV) (3). The oomycete *Phytophthora* (14), followed by *Hyaloperonospora* (8) and *Peronospora* (4), were the most covered. The insect genera *Helicoverpa* (4), *Nilaparvata* (3), *Spodoptera* (2), *Sogatella* (1), *Rhopalosiphum* (1), *Bemisia* (1), and *Aphidoidea* (1), and the nematode genera *Meloidogyne* (4) and *Heterodera* (1), were also observed in the studies (Figure 4).

As a result, the diseases most frequently covered were bacterial leaf blight (BLB), bacterial leaf streak (BLS) in rice, and bacterial spot in tomatoes. For fungi, brusone in rice, gray mold in tomatoes, and Fusarium wilt in various crops, among other diseases, were the most commonly observed (Figure 4).

### 3.4. Types of Explants

**Figure 5:**
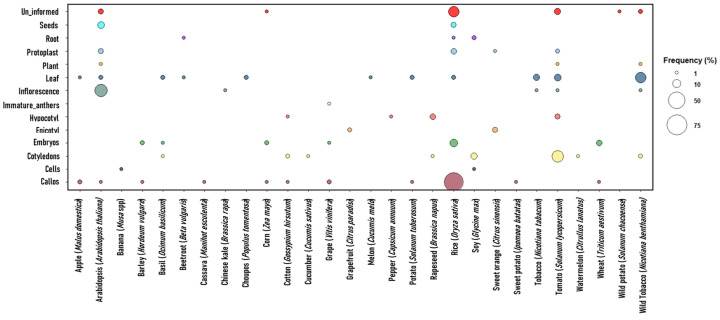
Explants used for the transformation of the different plant species covered in studies on gene editing via CRISPR/Cas for tolerance/resistance to biotic stress in the last 12 years. The colors of the circles represent each explant and the size of the circumference the frequency of each explant in different crops.

The explants used for plant transformation via CRISPR/Cas varied according to the plant species. Callus, cells, cotyledons, embryos, epicotyl, hypocotyl, anthers, inflorescence, leaf disks/leaves, plants, protoplasts, roots, and seeds were found as transforming materials (Figure 5).

For the rice crop, transformation via CRISPR/Cas was mainly performed using embryogenic calli, with a frequency of over 50%. Embryos, protoplasts, seeds, leaves, and roots were also used as transforming sources, but with a frequency of less than 10%. In tomatoes, the most commonly used explants were cotyledons, with a frequency of more than 10% of the studies performed on this crop, followed by leaves (<10%). In *Arabidopsis*, the inflorescence (>10%), seeds, protoplasts, leaves, and plant (<10%) were used as explants. In wild tobacco, the leaves (>10%), inflorescence, plant, and cotyledons (<10%) were used as the main transformation explants (Figure 5).

### 3.5. Plant Disease Resistance/Susceptibility Genes

**Figure 6:**
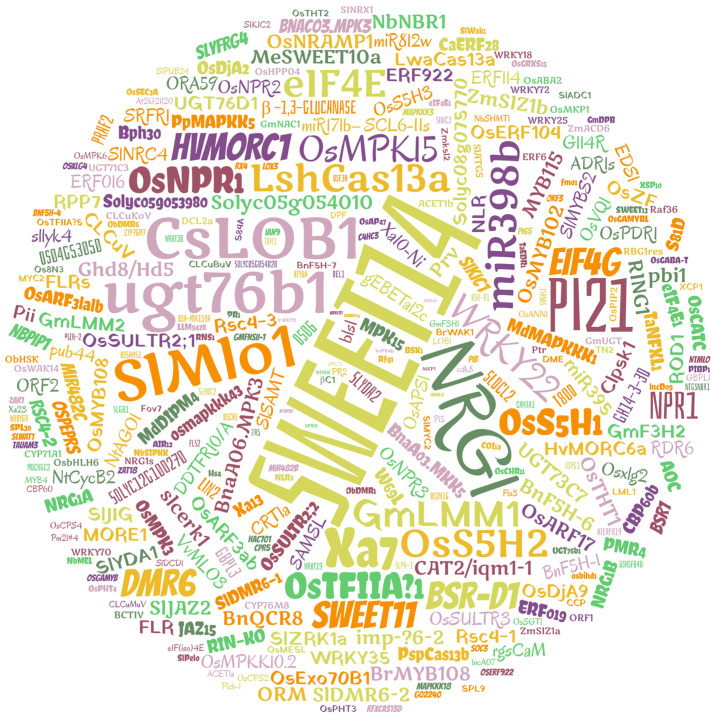
Word cloud of CRISPR/Cas edited genes in different plant species related to tolerance/resistance/susceptibility to biotic stresses.

A word cloud designed from the genes cited in the papers as potential targets for resistance/susceptibility to plant diseases. The sucrose efflux transporter gene (*SWEET14*) appears prominently in the center of the word cloud and was the most cited in the papers, especially those related to resistance to bacteria *Xoo* in rice, followed by N Requirement Gene 1 (*NRG1*), *Pi21* resistance genes, LateraL Organ Boundaries 1 (*CsLOB1*), Mildew resistance locus o 1 (*SlMlo1*), Dependent Glycosyl Transferases (*UGT76b1*), and the *Xa7* resistance gene. The gene families *WRKY* (14), *SWEET*/*OsSWEET* (12), *UGT* (7), *Xa* (7), and *Solyc* (5) have also been extensively studied. In the articles selected for this SR, 337 genes related to tolerance/resistance to biotic factors were covered; some papers used CRISPR/Cas technology to edit more than one gene (Figure 6).

### 3.6. Auxiliary Methods to CRISPR/Cas

**Figure 7:**
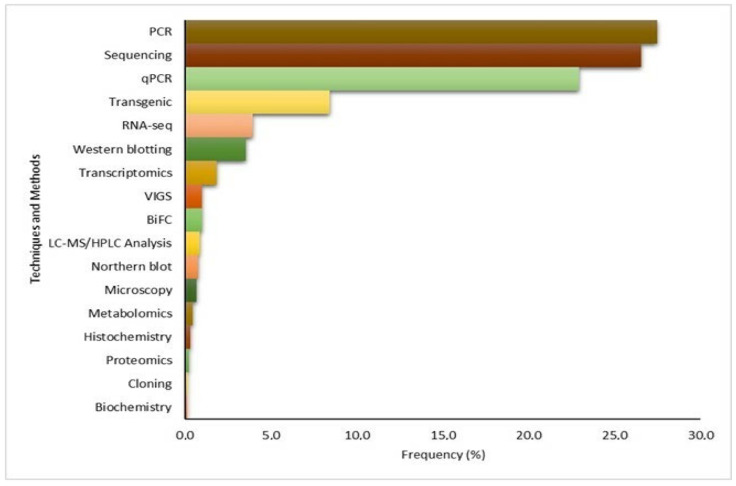
Auxiliary tools and analyses used with the CRISPR/Cas technique for validation and comparison between knockout with control and/or the overexpression of mutants identified in articles on tolerance/resistance to biotic stresses in the last 12 years.

The methodological strategies most used in the studies collected to validate and support the CRISPR/Cas tool were PCR (27.4%), sequencing (26.5%), qPCR (22.9%), transgenics (8.4%), RNA-seq (3.9%), Western blotting (3.5%), transcriptomics (1.8%), virus-induced gene silencing (VIGS) (1.0%), bimolecular fluorescence complementation (BiFC) assay (1.0%), LC-MS/HPLC liquid chromatography analysis (0.9%), Northern blot (0.7%), microscopy (0.6%), metabolomics (0.4%), histochemistry (0.3%), and proteomics (0.2%) (Figure 7). The other methods accounted for less than 0.1% of the studies.

PCR, sequencing, and qPCR techniques were mainly used to demonstrate the efficacy of the CRISPR/Cas tool and detect on- and off-target mutations.

**Figure 8:**
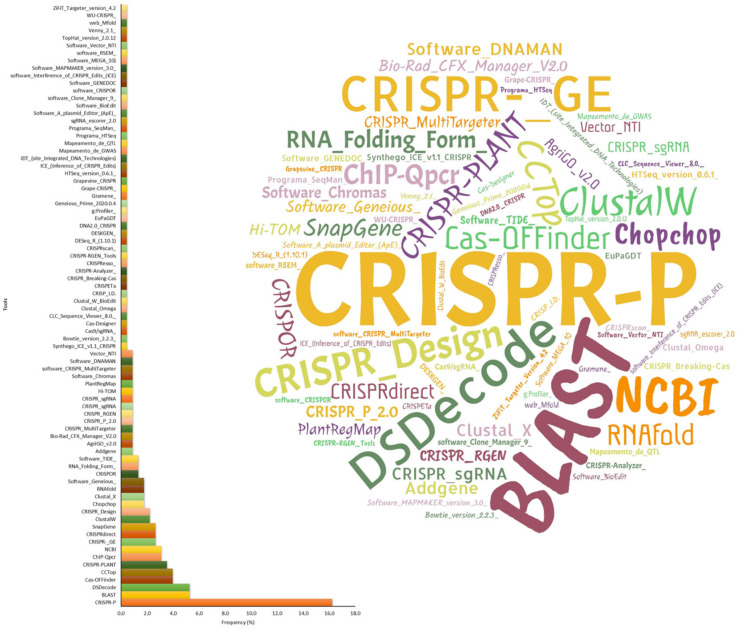
Frequency and word cloud of tools and software that help the CRISPR/Cas technique to search for specific target sites identified in studies on tolerance/resistance to biotic stresses in the last 12 years.

Certain types of software were also used to complement CRISPR/Cas-related analyses. The CRISPR-P version 2.0 software appears in 16.2% of the articles as an auxiliary method to CRISPR/Cas to predict target sites and/or mutations. Other widely used software/programs included BLAST, DSDecode, Cas-OFFinder, CCTop, CRISPR-PLANT, NCBI, CRISPR-GE, CRISPRdirect, SnapGene, ClustalW, CRISPR Design, CHOPCHOP, ClustalX, RNAfold, Geneious, CRISPOR, RNA Folding Form, and TIDE (Figure 8).

### 3.7. Use of CRISPR/Cas Technology

Most of the CRISPR/Cas methods used in the 296 studies selected for this SR had already been validated by other authors. The method used by Ma et al. (2015) [48], Xing et al. (2014) [49] and Wang et al. (2015) [50], and showed great reproducibility, being used in 24.7% of the studies (Table S2) to precisely edit plant genomes, deleting regions responsible for unwanted characteristics or inserting gain-of-function mutations.

For the CRISPR tool to be effective as Cas, endonuclease must be used. Of the studies collected, 98.3% (291) used Cas9 as an accessory to this editing system. Other endonucleases such as Cpf1, formerly known as Cas12a (2) and Cas13 (3), were also mentioned, but they were not very common.

Several vectors have been used to express Cas and/or single guide RNA (gRNA), but the most commonly cited is pCAMBIA and pYLCRISPR/Cas. The most widely used delivery method for introducing the gene of interest into plant cells was carried out by *Agrobacterium tumefaciens* (286) and *Agrobacterium rhizogenes* (6), occurring mainly via electroporation and heat shock.

### 3.8. Phenotypic Analysis and Characteristics Obtained after Mutation

**Figure 9:**
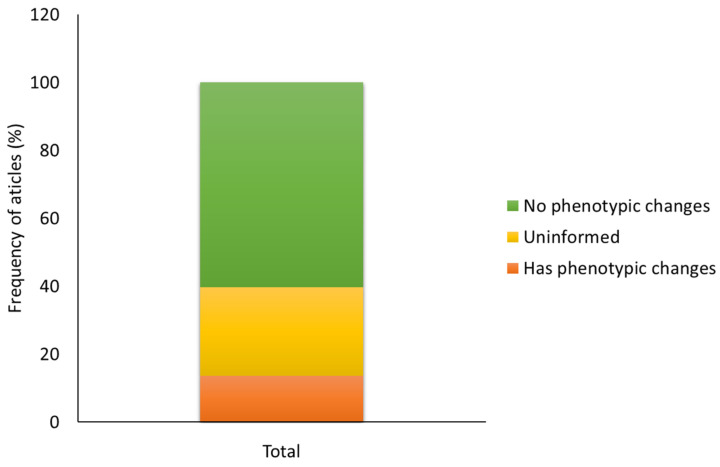
Frequency of articles that performed a phenotypic analysis of plants after mutation and the pathogen inoculation test.

Considering the agronomic characteristics and visible symptoms of the disease after mutation of the plants, 60.2% of the studies indicated that the phenotype was preserved, 13.7% inferred that the plants showed unusual characteristics after mutagenesis, such as dwarfism, albinism, and more aggressive symptoms of the disease, such as wider lesions than would be characteristic, and 26.1% of the articles did not perform this type of analysis or did not record having carried it out (Figure 9).

Greater resistance to plant diseases was observed in approximately 70% of the studies and higher plant susceptibility after gene mutation was noted in 28% of the studies, indicating that these genes are related to plant defense/immunity response (Table S2).

### 3.9. Sources of Bias in the Included Studies

To assess risk of bias in individual studies, an adaptation of the Cochrane risk of bias tool protocol was performed, which is composed of domains; according to the reviewers’ judgment, the study/outcome is classified as having a high, low or unclear risk of bias. The domains assigned to this SR are important and necessary questions in studies related to gene editing by CRISPR/Cas technology. Thus, questions such as “Was phenotypic analysis performed after mutation in the plant?”, “Was off-target analysis performed?” were used to classify the methodological quality of the selected articles. And three authors did these analyses independently to avoid potential biases.

**Figure 10:**
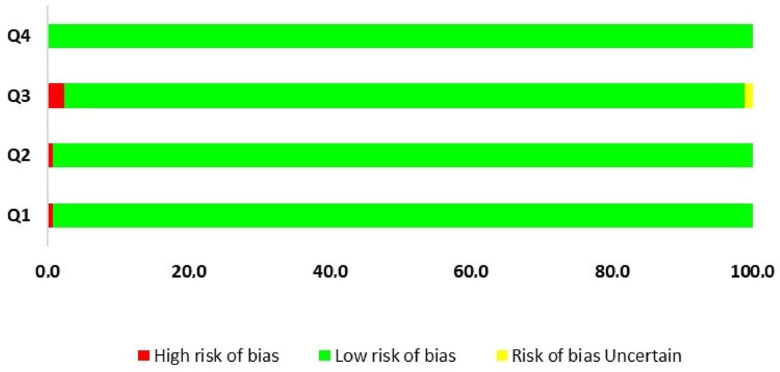
Risk of bias analysis based on the following questions: “Q1: Was off-target analysis performed? Q2: Was the pathogen inoculated? Q3: Was phenotypic analysis performed after mutation in the plant? Q4: Does the article answer at least 50% of the research questions?”.

Based on the classification defined for the risk of bias and the questions designed to measure the risk, it can be inferred that 98.6% of the articles presented a low risk of bias (Figure 10). Only six articles did not answer question 3 (“Was phenotypic analysis performed after mutation in the plant?”) and presented a high risk for this question. Three studies had an uncertain answer; however, the other questions were answered, which does not invalidate these studies from contributing to this SR. For question 1 (“Was off-target analysis performed?”), only two studies did not answer. The other questions were answered in full, confirming the good methodological and bibliographical quality of this study (Table S3).

## 4. Discussion

### 4.1. Bibliographic Survey

This SR presents a compilation of data extracted from articles carefully selected between 2013 and 2024, with the aim of expanding knowledge on the use of CRISPR/Cas technology in plant gene editing for resistance to biotic stresses. The application of the CRISPR/Cas system in plants began in 2013 [51,52,53,54]; however, until 2015, the works consisted mainly of preliminary studies and the validation of techniques and protocols. Literature reviews were rejected to avoid bias, and letters to the editor and non-peer-reviewed articles were also disregarded. For this reason, and to obtain more recent studies on the subject, articles from the last twelve years were considered.

The year 2021 saw the largest bibliographic contribution on the subject, which may be related to the increased demand for food in the world and the negative effects of the COVID-19 pandemic [55], which led to an 18% increase in production in 2021 and an 11% increase in 2022 [56], stimulating agribusiness and studies focused on the genetic improvement of crops in order to minimize food shortages. The amount of data obtained on the subject in recent years reveals its importance and the need for investment in this area of research aiming to provide returns for the population and rural producers. Furthermore, these data reveal that technology is evolving rapidly and could contribute to overcoming food shortages for exponentially growing populations [57].

The biometric analysis demonstrated that the keywords “disease resistance” or “CRISPR/Cas9” present in the search string are also the most cited words in the selected articles and indicate that, over the last twelve years, more than 5000 studies have focused on this topic.

Keywords such as “viral resistance”, “DNA”, “genomics”, “oomycetes”, “soybean”, and “*Sclerorotinia sclerotiorum*” appear in isolation despite being related to the topic; this is because such words are found mainly in the body of the text and not in the titles, abstracts, and keywords of the selected articles.

The authors who have produced the most studies on the subject are from research institutions located mainly in China and the USA. The Rice Research Institute and Key Lab for Major Crop Diseases located at Sichuan Agricultural University in China is responsible for a major contribution to gene-editing work using CRISPR [58,59,60].

### 4.2. Study Sites and Edited Crops

Most of the studies included in this SR originated in China (140), which is in line with the data on agricultural production. Despite having less than 10% of the world’s productive land, the country ranks first in the production of cereals, cotton, fruit, vegetables, meat, poultry, and fishery products, as well as accounting for 25% of the world’s grain production [61]. This makes the country a major contributor to crop improvement studies using the CRISPR/Cas tool.

The studies performed in the USA were also representative (27). The country is the third largest food producer in the world and the first when it comes to exporting corn and soybeans, the main agricultural commodities [61]. Countries such as Germany, South Korea, Canada, Pakistan, Spain, and India have also contributed to studies on the subject.

Rice is the second-most-produced food crop in the world and the first-most-cultivated in China, which accounts for 30% of world production [61]. It is a monocot considered a model, because its genome is small and easy to manipulate when compared to other crops, which justifies the large number of studies (107) using CRISPR/Cas technology as a gene-editing tool for improving this crop [62,63,64,65].

In addition to rice, 27 other plant species have been covered in gene-editing studies in this SR. Tomato is the second-most-cited crop (47 articles) and the sixth most important crop economically, with a production of more than 100 million tons per year [61]. The model crops, *Arabidopsis thaliana* and *Nicotiana* sp., were also well cited in the selected papers; this may be related to the large amount of information already validated on these species and because their genomes have already been sequenced [66,67,68,69].

### 4.3. Biotic Stresses

Biotic stressors such as pathogens, insect pests, and weeds reduce the yield and quality of agricultural production. In high-yielding crops such as wheat, rice, corn, potatoes, and soybeans, losses can range from 17.2% in potatoes to 30% in rice [70]. Several diseases affect rice cultivation. Bacterial leaf blight (BLB), caused by *Xanthomonas oryzae* pv. *oryzae* (*Xoo*), is considered one of the most important bacterial diseases of rice. Irrigated or rainfed areas are common for growing this species and favor the development of the disease due to the abundance of water facilitating the dispersion of the pathogen, or through the high availability of nitrogen [62,71,72,73,74,75].

Bacterial leaf streak of rice caused by *X. oryzae* pv. *oryzicola* (*Xoc*) is also another disease that has been widely covered in the studies collected [76,77,78]. The genus *Xanthomonas* has been the most studied in gene editing via CRISPR/Cas in the last twelve years [75]. The studies seek to understand the mechanisms involved in plant defense against pathogens in order to make them resistant/tolerant to diseases.

Brusone is the main fungal disease of rice, caused by *Magnaporthe oryzae*, which establishes itself in the plant under favorable environmental conditions and causes damage to grain quality, plant height, and the number of tillers [79]. Rice is grown and consumed worldwide and is a staple food for around 2.5 billion people [61], so it is necessary to understand the biology of these pathogens to develop strategies to control these diseases.

*Pseudomonas syringae* was another pathogen that was mentioned frequently [80,81,82,83]. This bacterium is found in a wide variety of plants and penetrates host tissues through lesions or structures such as stomata [84]. The species has been widely used to elucidate questions about plant immunity and bacterial pathogenesis. In the selected articles, the bacterium is mainly present in studies with the model plant *A. thaliana* [66,85].

Tomatoes are an economically essential vegetable worldwide and their production is also threatened by many pathogens [86,87,88]. Gray mold, caused by *Botrytis cinerea*, rarely occurs in the field; however, in protected environments, humidity becomes a problem, favoring the development of the fungus, which infects the plant through wounds and causes the rapid rotting of the fruit, resulting in harvest losses. Other biotic agents have also been addressed in the studies, such as fungi (such as *Fusarium*), CMV viruses, CLCuVs, and the *Phytophthora* oomycete.

### 4.4. Types of Explants

The genetic transformation of plants is based on the insertion of transgenes into totipotent plant cells, which then regenerate into fertile plants. Small fragments of living tissue isolated from a plant specimen, called explants, are used [89]. The explants used for transformation via *Agrobacterium* or bioballistics can vary depending on the plant species, including calli, embryos, protoplasts, inflorescence, leaves, hypocotyls, epicotyls, and cotyledons [2,62,90,91,92,93,94,95].

When transformation occurs by electroporation, protoplasts undergo membrane destabilization after being subjected to high voltage, resulting in temporary pores in the cell membrane, allowing for the influx of DNA molecules that will integrate into the genome of the species to be mutated [96]. This type of method requires plants to be obtained entirely from protoplasts, which requires mastery of the production and regeneration of this type of explant, which is still a challenge in tissue culture [97].

The vast majority of the studies collected for this research used embryogenic calli and leaves as explants for various plant crops. In rice, the use of calli as an explant source is predominant [62,74,98,99,100]. Calli are formed practically from any fragment of the plant; in rice, seeds are commonly used to induce calli, which grow slowly as an amorphous cell mass through stimuli supplemented with specific phytohormones [89].

In tomatoes, transformations have been carried out mainly from cotyledon and leaf explants [81,93,101]. Most tissue culture tests in this species have been performed to achieve organogenesis over somatic embryogenesis [102]. In studies performed by Costa et al. (2000) [103], the tomato varieties ‘IPA-5’ and ‘IPA-6’ demonstrated favorable regeneration capacity (97 and 80%, respectively) from cotyledons when inserted into a supplemented Murashige and Skoog medium.

Studies with *A. thaliana* have mainly used flowers/inflorescences as a source of explants [92,104,105,106]. The floral immersion method is considered simple, fast, and efficient, and consists of immersing developing floral tissues in a solution containing *Agrobacterium tumefaciens*, sucrose, and detergent to transform the plants [107,108].

In tobacco, leaves were the most commonly used explants [68,109,110]. In direct somatic embryogenesis tests, using leaf tissue explants from different tobacco genotypes, different *Agrobacterium* strains, and different transformation methods, the transformation and regeneration rates varied [111,112]. The success of the transformation system involves the integration of the DNA into the host genome, the expression, inheritance, and stability of the exogenous DNA, as well as the regeneration of explants that depend largely on the genotype, origin, and age of the explant and plant growth regulators used to supplement culture media [113].

### 4.5. Plant Disease Resistance/Susceptibility Genes

The gene that stood out in CRISPR/Cas studies for resistance to biotic stresses was *SWEET14* (Figure 6). *SWEET* genes encode sugar transporter proteins and often function as susceptibility (S) genes, the recessive alleles of which provide resistance [114]. This gene has been extensively studied and reviewed in studies involving the bacterial pathogen *Xoo*, which causes bacterial rust in rice [114,115]. In the context of plant–pathogen interaction, transcription activator-like effectors (TALEs) of the pathogen function in diverting the nutritional resources of rice, inducing the expression of *OsSWEET14* and thus causing susceptibility [72,114]. The activation of *SWEET14* by the pathogen results in an increase in the amount of sucrose available in the phloem apoplast, providing a source of nutrition for the pathogen promoting its proliferation [116].

The main strategy when using CRISPR/Cas9 in relation to the *SWEET14* gene is to mutate the coding region of *OsSWEET14* to test whether its disruption will result in broad-spectrum resistance to *Xoo* strains in rice [72,117] or the disruption of the TALE-binding elements of *Xoo* in rice harboring the recessive resistance allele in order to defuse the arms race between the effectors of the pathogen and their host targets [26,118,119]. Inhibition or *SWEET14* editing can reduce the plant’s susceptibility to the pathogen, a potential strategy for the development of resistant cultivars.

Other genes, such as *NRG1*, *Pi21* resistance genes, *CsLOB1*, *SlMlo1*, dependent glycosyl transferases (*UGT76b1*), and the *Xa7* resistance gene, were also reported with considerable frequency in the studies (Figure 6). The *NRG1* genes are close homologs of the Activated Disease Resistance 1 family of leucine-rich repeat domain proteins (NLRs), the function of which is still unclear, so some studies have reported their functional analysis through CRISPR/Cas9 in *Arabidopsis* [120,121,122]. The *Pi21* gene belongs to the set of R genes that encode NLRs. It is resistant to rice brusone and is, therefore, the target of CRISPR/Cas9 rice-breeding programs to obtain mutant varieties [75,123,124,125].

Plants can prevent pathogen attacks through induced systemic resistance (ISR) and acquired systemic resistance (SAR). What differentiates them are the types of induction in the plant. SAR is activated through disease-causing organisms and relies on salicylic acid (SA) and genes, whereas beneficial microbes induce ISR and are independent of SA [126]. The two forms of resistance are activated from different defense signals when the plant is attacked by pathogens [127].

### 4.6. CRISPR/Cas Technology for Gene Editing

This SR sought to identify the most commonly used protocols for gene editing via CRISPR over the last twelve years. Among the editing methods used, the protocols proposed by Ma et al. (2015) [48], Xing et al. (2014) [49] and Wang et al. (2015) [50] were the most cited, respectively. The three protocols seek to edit various target genes in dicots and monocots using a multiplexing system, using one to several binary vectors and the Cas9 endonuclease. These results corroborate the findings of [22] in a systematic review of gene editing using CRISPR technology to edit genes tolerant to abiotic stresses.

Different CRISPR/Cas systems have been widely used to generate DSBs at target genomic sites in various plant species. Among the two classes of CRISPR immune systems, Class 2 is simpler than Class 1 and therefore easier to use for the development of genome editing tools [128]. Thus, three Class 2 effectors, Cas9, Cas12, and Cas13, have been extensively used for targeted DNA and RNA cleavage. The Cas9 endonuclease was the most widely used in the articles in this SR (259 studies), followed by Cas13 (3), and Cas12 (2). The effectors Cas9 and Cas12 are DNA-directed endonucleases, while Cas13 is an RNA-directed endonuclease [129].

As evidenced by Jinek et al. (2012) [14], Cas9 nucleases are guided by an RNA hybrid consisting of a crRNA and a tracrRNA. However, most Cas9 genome editing applications use an sgRNA that is designed by fusing crRNA and tracrRNA into a single RNA molecule for Cas9 to cleave DNA [130,131]. Normally, CRISPR/Cas9 requires a target site of 17 to 20 bp directly adjacent to a 5′-NGG PAM sequence (motif adjacent to the protospacer) to be effectively recognized by sgRNA [15,132]. Several authors have used Cas9 [68,133,134,135,136], and although several Cas9 orthologs have been discovered [137], Cas9 from *Streptococcus pyogenes* (SpCas9) is the nuclease that has been used the most for different genome manipulation experiments due to its high efficiency and simple NGG PAM sequence requirements [129].

The Cas12 endonuclease was identified in this SR with the aim of knocking out Xa13 [138] and PRAF2 [28] to improve resistance to bacterial rust caused by *Xanthomonas oryzae* pv. *Oryzae*. Cas12 is a class II type V endonuclease that was developed from *Prevotella* and *Francisella* [139]; it cleaves at a distal position of the PAM, generating a staggered break of the DNA double-strand, and recognizes a PAM region rich in T 5′-TTN-3′ [140] and proved to be an efficient alternative in editing these genes. Cas13 cleaves single-stranded RNA [141], and in the studies observed it was used to interfere against RNA viruses in plants, also presenting itself as a viable alternative to the use of Cas9 [142,143,144].

Cas9 and gRNA are regulated by appropriate promoters within a vector. The cauliflower mosaic virus (CaMV35S) is a constitutive promoter widely used for its strong expression in various plant tissues, being effective for mutations throughout the organism. The ubiquitin promoter (UBI), also constitutive and commonly used in monocots, has efficient and stable expression, especially in recalcitrant cultures. In addition, specific tissue promoters can also be used to induce mutations in plants. These allow for more controlled editing, limiting the expression of the system to specific sites, which reduces off-target effects, but can make mutations in the whole organism less efficient. The choice of promoter is crucial for the efficiency of the mutation due to to the objectives of gene editing, such as the need for localization or plant-wide expression, species compatibility, expression and efficiency, and the risks of off-site effects [145,146,147].

Several vectors have been used to express Cas and/or sgRNA, among the most cited being pCAMBIA (46) [63,73,148] a popular vector due to its easy handling, stability, and the existence of a variety of selection and reporter genes [149], and the pYLCRISPR/Cas9 vector (40) [150,151], which is a CRISPR/Cas9 system efficient in multi-locus gene knockout [48]. Other vectors, such as pHEE401E [152], also had considerable frequencies (Table S2). The most widely used delivery method for introducing the gene of interest into plant cells was carried out by *Agrobacterium tumefaciens* (286) and *Agrobacterium rhizogenes* (6). This is considered a powerful tool for delivering genes of interest to a host plant due to the efficiency of transformation, the low operating cost, and the simplicity of the transformation and selection protocols [153].

Although *Agrobacterium*-mediated delivery is very efficient, it also has some disadvantages, such as the need for long periods of tissue culture to recover transgenic plants, the low frequency of stably transformed plants, the narrow range of genotypes within a crop species that can be transformed, and the limitations of the host range of certain *Agrobacterium* species [154]. The delivery of CRISPR/Cas reagents to plants can be carried out by several methods. The most common in addition to *Agrobacterium tumefaciens*-mediated transformation include particle bombardment (biobalistics) and protoplast transfection [155,156].

Particle bombardment is useful for recalcitrant plant species, but it can cause physical damage to cells and random DNA integrations. Protoplast transfection, on the other hand, allows for the delivery of ribonucleoproteins (RNPs), reducing the risk of exogenous DNA integration, but the regeneration of complete plants from protoplasts can be challenging in some cultures [155,156]. Additional delivery methods of the CRISPR/Cas system, such as the use of nanoparticles and pollen magnetofection, can be an alternative for more precise and efficient delivery [157].

### 4.7. Auxiliary Methods to CRISPR/Cas

The main methodological strategies used in the studies collected to validate and support the CRISPR/Cas tool were PCR, sequencing, and qPCR techniques (Figure 7); these were mainly used to prove the efficacy of CRISPR/Cas-mediated editing and detect on- and off-target mutations (Figure 7). The PCR technique is an essential tool in molecular biology that allows for the amplification of nucleic acid sequences (DNA and RNA) through repetitive cycles in vitro, simulating what occurs in vivo during DNA replication [158].

PCR followed by sequencing has been reported in many studies; however, Zischewski et al. [159] highlight that a disadvantage of screening only potential pre-selected off-target sequences is the risk of overlooking mutations at other loci in the plant genome. In contrast, the use of the unbiased whole-genome-sequencing approach is the most common detection method in plants, allowing for the identification of off-target effects in a less restricted way [160].

Different prediction software were also used to detect off-target effects (Figure 8). The CRISPR-P software was reported in 16.2% of the articles as an auxiliary method to CRISPR/Cas, aimed at predicting target sites and/or mutations. Other software/programs, such as BLAST, DSDecode, Cas-OFFinder, CCTop, CRISPR-PLANT, and CHOPCHOP, also had considerable frequencies. A major concern in CRISPR/Cas9 system applications is its off-target effects that occur when Cas9 acts on untargeted genomic sites and creates cleavages that can lead to adverse outcomes [161].

The tools identified in this SR aid in silico prediction and are generally free online software that can be properly accessed via the Internet. The prediction algorithms of these software are mainly based on sgRNA sequences, so the results of these methods are generally biased toward sgRNA-dependent off-target effects. For epigenetics and chromatin organization experiments, off-target prediction by these in silico tools needs additional experimental validation [161].

### 4.8. Phenotypic Analysis and Characteristics Obtained after Mutation

In 60.2% of the articles, the phenotype was preserved, with no unusual or unexpected characteristics occurring after mutagenesis. Sixty-one percent exhibited greater resistance to plant diseases and 29% greater susceptibility after editing (Figure 9). This is because most studies are focused on knocking out/silencing genes or knocking in/overexpressing a gene to study and demonstrate its functions. Thus, the technique that cuts double-stranded DNA and generates a DSB will be repaired by the NHEJ repair mechanism; this can be carried out for a specific and individual gene without other side effects [162].

In other articles, the CRISPR/Cas technique has been used to knock-in the overexpression of an individual gene. In this sense, it is possible to edit the genome by cutting the DNA sequence at a specific site, and then, through HDR, a foreign DNA sequence (target gene) will be inserted at this cleavage site [162]. In this way, position effects can be avoided because CRISPR/Cas can be used to precisely insert a foreign gene into a specific location within a genome without interrupting other genes.

In this sense, the overexpression of the OsbHLH6 gene in transgenic rice plants caused responsive gene expression to jasmonic acid and increased susceptibility to the pathogen *Magnaporthe oryzae* [63]. Similarly, the overexpression of the GmLMM1 gene in Nicotiana benthamiana severely suppressed the production of reactive oxygen species triggered by microbe-associated molecules (bacterial flg22) and the pattern-induced cell death of the oomycete *Phytophthora sojae* [163].

Thus, the use of the CRISPR/Cas technique associated with gene knockout/silencing or gene knock-in/overexpression has contributed to the elucidation of various plant–pathogen interaction pathways in many pathosystems, without causing unwanted phenotypic changes, such as citrus canker caused by *Xanthomonas citri* subsp. *citri* in citrus [2], BLS of rice caused by *Xoc* and *Xanthomonas campestris* pv. *campestris* [73,164,165], *Phytophthora sojae* in soybeans [144], and *Botrytis cinerea* in tomatoes [81].

### 4.9. Sources of Bias in the Included Studies

The aim of SRs is to gather and synthesize data on a given topic that meets pre-established eligibility criteria and methods are used to reduce the chances of data bias [166]. The Cochrane Collaboration Tool was developed to assess the risk of bias of the studies to be included in the SRs and is widely used in health studies [47], which is why the method was adapted to the needs of this SR.

The tool aims to make the process clearer and more precise, free from errors that compromise the quality of the research. Therefore, possible limitations of the primary studies must be carefully assessed so that the results and conclusions obtained are reliable. It is not possible to determine the “quality” of a study without any kind of criteria; it is necessary to observe the design, the conducting of the research, and the analysis and presentation of the results so that the studies are not underestimated or overestimated [47,167].

In order to minimize errors in the choice of studies collected for this SR, inclusion/exclusion criteria, the PRISMA checklist, and questions on the topic (Table 3) were used to confirm whether the use of CRISPR/Cas technology was efficient in gene editing through off-target analysis. Inoculation tests of the pathogen and phenotypic analysis were also considered, as well as articles that answered at least 50% of the research questions (Table 1).

Literature reviews were excluded from the research, as many papers are cited repeatedly in the reviews, overestimating the data. Manuscripts that did not answer at least 75% of the risk-of-bias questions were considered high-risk and were not included in this SR. Only nine articles presented a risk equal to 25% for not answering one of the four questions, which is considered a low risk of bias, and two articles presented moderate risk, which means they answered only 50% of the questions. The articles selected for this SR are highly qualified and the methodologies used are reliable.

## 5. Final Considerations and Future Perspectives

The growing demand for food is a challenge for society in the face of population growth, changes in consumption patterns, environmental changes, and dealing with pathogens that cause plant diseases and pests. Meeting this demand is based on the need to guarantee global food security.

Biotic and abiotic stresses cause major losses in agricultural production, which calls for novel strategies to subsidize plant tolerance, as conventional practices are insufficient to meet the current and future food needs of the population. The use of the CRISPR/Cas tool can accelerate plant breeding by rapidly modifying genomes in a predictable and accurate way. Due to its efficiency, simplicity, and versatility, CRISPR/Cas has become a popular tool for genome editing and has been widely used in improving the resistance of various crops [57]. The development of disease-resistant varieties with good yields and quality is a fundamental strategy to guarantee global food security and generate employment and income for farmers.

This SR included 296 papers in which plant genes were edited via CRISPR/Cas to confer resistance to plant diseases and pests. We identified that Cas9 endonuclease is widely used in studies; however, this is not the only “molecular scissors” that can help the CRISPR editing system; the use of other enzymes such as Cpf1 (Cas12a), and Cas 13 has been reported in CRISPR studies for editing genes related to plant resistance and could be applied more frequently in future studies.

Genes related to tolerance/resistance to biotic stresses were identified in this SR and the CRISPR/Cas system can be used for gene knockout, gene insertion and gene replacement, resulting in the loss of function, knockdown or activation of mutants, which can lead to the generation of tolerant/resistant plants to the various pathogens. However, some issues are still far from being clarified and serve as a starting point for future studies, such as the fact that the main genes that control important traits of crops have not been identified, which limits the application of CRISPR/Cas in plant breeding; and pathogens continue to modify their genome through evolution to break the already available resistance gained by editing the CRISPR/Cas gene. Thus, it is necessary to design new variants in a short period of time and insert them into the plants. In addition, many genes are represented by multigene families, making it difficult to produce a resistance phenotype by eliminating a single gene, and it is necessary to develop more precise CRISPR/Cas tools to perform multiplex genome editing.

Regarding the methods used for editing, gRNAs were designed with different target sequences to direct Cas9 to specific corresponding sites; however, proper care is important when designing gRNAs, as unwanted targets are a major limitation, and to reduce these challenges, tools and software such as CRISPR-P, CRISPR-GE, BLAST, among others, are used. Among the methods used for mutation detection, PCR and sequencing are the most reported methods that can detect unwanted targets. Explant regeneration in most plants is still a challenge because it is labor-intensive and poses a limitation in CRISPR/Cas-based gene editing.

The information provided in this SR was based on articles with methodological quality confirmed by a risk of bias analysis, which determined that most of the included studies were at low risk of bias. Among the most-studied crops, rice, tomatoes, and the model plant *Arabidopsis thaliana* stand out. Among the most studied genera of biotic agents are *Xanthomonas*, *Magnaporthe*, *Phytophthora* and cucumber mosaic, belonging to the group of bacteria, fungi, oomycete and viruses, respectively.

Although the use of CRISPR/Cas technology has revolutionized plant breeding in recent years, there are still many challenges to be overcome; its off-target alterations are the main bioethical concern, namely whether they can lead to ecological imbalance, genetic drift, fatal diseases, or a chimeric phenotype in animals or even in humans. Another concern is whether GMOs produced by CRISPR/Cas9 can change the natural ecosystem by changing the mating potential of living organisms. Agricultural foods produced by CRISPR also face the same challenges as GMOs and may be prevented from being consumed in some countries. Despite these concerns, plants developed CRISPR/Cas can also become safe and GMO-free by using ribonucleoproteins (RNPs), i.e., without exogenous DNA. This will also help overcome the hurdles scientists face in commercializing biotech crops. To date, around 128 plant cultivars such as corn, soybeans, cotton, wheat, and sugar cane have been genetically edited, mainly for resistance to insects and/or herbicides, and have been approved by the National Technical Biosafety Commission [168].

Studies on gene editing with CRISPR/Cas for resistance to biotic agents are only beginning. The results obtained so far not only show that this technology offers precise modifications to the plant genome and has been successfully used to confer resistance to diseases and pests, but are also essential mainly to understand the function of genes related to various pathways of plant–pathogen interaction.

## Figures and Tables

**Table 1:** Description of the PICOS strategy used to develop the RS research questions on the use of CRISPR/Cas technology to edit tolerance genes/resistance to biotic stresses from studies published in the last 12 years.

Description	Abbreviation	Components of the Question
Population	P	Agricultural varieties under biotic stresses
Interest/Intervention	I	Gene editing in plants using CRISPR/Cas technology for disease resistance
Comparison	C	Plant breeding methods
Outcome	O	Editing genes that confer resistance to biotic stresses in plants
Study type	S	Scientific articles and literature reviews

**Table 2:** Guiding questions for this SR on the use of CRISPR/Cas technology to edit tolerance genes/resistance to biotic stresses from studies published in the last 12 years.

Research Questions
1. In which country was the study performed?
2. What culture is the article about?
3. Which biotic agent is addressed in the study?
4. Which genes are reportedly associated with disease and pest resistance in plants?
5. Which nuclease is used in conjunction with the CRISPR tool?
6. What methodology is used to use CRISPR?
7. What method is used to prove the effectiveness of the tool?
8. What techniques/tools are associated with CRISPR/Cas9?
9. What transformation method was used?
10. Which explant was used to transform the plants?
11. What are the main vectors used to express Cas9 and/or gRNA in plants?
12. Were any unusual phenotypic characteristics observed in the plants after genetic transformation? Which ones?
13. What is the characteristic obtained after mutating the plant?

**Table 3:** Questions to evaluate the methodological quality of the articles included in the SR on the use of CRISPR/Cas technology to edit tolerance genes/resistance to biotic stresses from studies published in the last 12 years.

Risk of Bias
1. Has off-target analysis been performed?
2. Has the pathogen been inoculated?
3. Has phenotypic analysis been performed after mutation in the plant?
4. Does the article answer at least 50% of the research questions?

## Data Availability

Not applicable.

## Supplementary Materials

The following supporting information can be downloaded at: https://www.mdpi.com/article/10.3390/cimb46100659/s1, Table S1: The selection of articles on the use of CRISPR/Cas technology associated with biotic stress tolerance in plants in the last twelve years. Table S2: Methods used for genome editing based on CRISPR/Cas technology and characteristics obtained by the plant after mutation in studies on biotic stresses in the last twelve years. Table S3: An assessment of the risk of bias of 296 articles selected for a systematic review on CRISPR/Cas technology used in gene editing in biotic stress tolerance published in the last twelve years. References [169,170,171,172,173,174,175,176,177,178,179,180,181,182,183,184,185,186,187,188,189,190,191,192,193,194,195,196,197,198,199,200,201,202,203,204,205,206,207,208,209,210,211,212,213,214,215,216,217,218,219,220,221,222,223,224,225,226,227,228,229,230,231,232,233,234,235,236,237,238,239,240,241,242,243,244,245,246,247,248,249,250,251,252,253,254,255,256,257,258,259,260,261,262,263,264,265,266,267,268,269,270,271,272,273,274,275,276,277,278,279,280,281,282,283,284,285,286,287,288,289,290,291,292,293,294,295,296,297,298,299,300,301,302,303,304,305,306,307,308,309,310,311,312,313,314,315,316,317,318,319,320,321,322,323,324,325,326,327,328,329,330,331,332,333,334,335,336,337,338,339,340,341,342,343,344,345,346,347,348,349,350,351,352,353,354,355,356,357,358,359,360,361,362,363,364,365,366,367,368,369,370,371,372,373,374,375,376,377,378,379,380,381,382,383,384] are cited in the Supplementary Materials.
